# Topical green tea formulation with anti-hemorrhagic and antibacterial effects

**DOI:** 10.22038/ijbms.2020.41397.9782

**Published:** 2020-08

**Authors:** Fatemeh Kalalinia, Nafise Amiri, Niloufar Mehrvarzian, Bibi Sedigheh Fazly Bazzaz, Mehrdad Iranshahi, Azadeh Shahroodi, Sepideh Arabzadeh, Mohammadreza Abbaspour, Shapour Badiee Aaval, Jebrail Movaffagh

**Affiliations:** 1 Biotechnology Research Center, Pharmaceutical Technology Institute, Mashhad University of Medical Sciences, Mashhad, Iran; 2 Department of Pharmaceutical Nanotechnology, School of Pharmacy, Mashhad University of Medical Sciences, Mashhad, Iran; 3 School of Pharmacy, Mashhad University of Medical Sciences, Mashhad, Iran; 4 Targeted Drug Delivery Research Center, Pharmaceutical Technology Institute, Mashhad University of Medical Sciences, Mashhad, Iran; 5 Department of Pharmaceutics, School of Pharmacy, Mashhad University of Medical Sciences, Mashhad, Iran; 6 Complementary Medicine Research Center, Faculty of Traditional Medicine, Mashhad University of Medical Sciences, Mashhad, lran

**Keywords:** Bleeding, Green tea, Hemostasis, Polyvinyl alcohol, Tannin, Topical formulation

## Abstract

**Objective(s):**

Potentially preventable death from uncontrolled hemorrhage clearly indicates the importance of simple, fast and efficient ways to achieving hemostasis. The aim of this study was to develop a topical formulation of green tea extract for reducing bleeding that can be helpful in hemorrhage control.

**Materials and Methods:**

Hydroalcoholic extract of green tea was isolated from *Camellia sinensis* and formulated in polyvinyl alcohol (PVA) to achieve two concentrations of 2% and 4% v/v. Folin-Ciocalteau assay was used to determine the total amount of tannins in extract. Rheological behavior of solutions was investigated by measuring viscosity at shear rates of 0–200 sec^−1^. Quantitative and qualitative microbial limit tests and minimum inhibitory concentration (MIC) assay were done. The effect of formulations on bleeding time was evaluated in an animal model.

**Results:**

The total amount of tannin in green tea extract was 3.8% w/w and addition of green tea significantly increased the viscosity of PVA. The results of MIC assay showed that PVA could not inhibit the growth of bacteria, while, 716 µg/ml of green tea and 2860 µg/ml of green tea/PVA 4% inhibited the growth of *Staphylococcus aureus *and *Pseudomonas aeruginosa*. In an animal study both 2% and 4% formulations were able to stop hemorrhage approximately at an equal time compared with tranexamic acid (TXA) 50 mg/ml as a control and the lowest bleeding time was 6.4±0.51 sec for green tea/PVA 4%.

**Conclusion:**

Based on our results, the topical formulation of green tea extract in PVA has a great potential for anti-hemorrhage applications.

## Introduction

Uncontrolled hemorrhage is one of the major causes of death in different injuries, viral hemorrhagic (dengue, Ebola hemorrhagic fever, etc.), and some hereditary diseases (like hemophilia, Von Willebrand, etc.) (1-3). Therefore, rapid and successful control of bleeding can be lifesaving. Although normal homeostatic system tries to maintain circulation after severe trauma or vascular surgery, severe blood loss in these situations leads to extreme challenges that may lead to death (3). On the other hand, wound infections are another critical problem facing trauma care that should be controlled (2). Therefore, controlling hemorrhage with different techniques, devices, and drugs is very important and should be improved to reduce trauma damages (2, 3). 

Tea, the most common drink after water worldwide (4), is an infusion of the leaves of the *Camellia sinensis* plant (5). It is categorized as green tea, oolong tea, or black tea based on the amount of their oxidized substances. The manufacturing process of green tea prevents the oxidation of green leaf substances (6). Different studies have shown that green tea has many therapeutic benefits such as significant antioxidant, anti-inflammatory, anti-carcinogenic, antibacterial, and neuroprotective properties (5, 7, 8). Green tea contains several components like polyphenols (include catechins), caffeine, theanine, proteins, vitamins, minerals, and pigments (8). Catechins are a type of polyphenol and are the main astringency component in green tea, long known as tannins and present to the extent of 8–15% in the dry tea leaf (9). Tannins give tea many of its great properties like anti-oxidative benefits. They can also affect hemostasis, reduce bleeding time, and promote the wound healing process (10, 11). 

Polyvinyl alcohol (PVA) ((C_2_H_4_O)_n_) is a water-soluble synthetic polymer (12). Due to its desirable properties like excellent thermo-stability, chemical resistance, film forming ability, biodegradability, biocompatibility, and low toxicity, PVA is one of the best known and most commonly used excipients in pharmaceutical sciences (13-15). 

Based on the importance of decreasing hemorrhage morbidity and mortality in combination with decreasing the risk of infections, in this study we designed an effective and low price topical formulation of green tea extract in PVA, characterized the formulations, and investigated the impact of formulations on bleeding time in an animal model.

## Materials and Methods

***Chemicals and reagents***

The green tea leaves were obtained from Golestan Co. (Iran). Polyvinyl alcohol (PVA) and polyvinylpyrrolidone (PVP) were purchased from Fluka (Germany). Tranexamic acid was from Caspian Tamin Pharmaceutical Co. (Iran), and ketamine and xylazine were from Alfasan (The Netherlands). Soybean casein digest broth (SCDB), cetrimide, and Vogel-Johnson agar were purchased from Himedia (India) and tetrathionate broth was from Gibco (Germany). tannic acid, Folin-Ciocalteu reagent, Sabouraud dextrose broth (SDB), Lactose broth (LB), MacConkey agar and other materials were obtained from Merk (Germany) unless otherwise noted. 

***Preparation of green tea hydroalcoholic extract***

Dried green tea leaves were powdered, then 200 g of the powder was soaked in 4000 ml of ethanol 70% (v/v) aqueous solution and shaken for 48 hr. The extract was then filtered with paper filter grade 1 (pore size of 11 µm) and the solvent was removed in a vacuum evaporator to obtain the concentrated extract. This concentrated extract was examined for tannin amounts and used for preparation of 2% ,4% (v/v), and other concentrations of green tea solution in the next steps.

***Determination of total tannins content in the green tea extract***

The Folin-Ciocalteau reagent was used to determine the total amount of tannins in green tea extract (16, 17). First, 100 mg of polyvinylpyrrolidone (PVP) was diluted with distilled water in 1:1 ratio to achieve 1 mg/ml PVP solution and then mixed with 1 ml of concentrated extract (18). The mixture was stirred for 15 min at 4 ^°^C and centrifuged at 2000 g for 10 min. The lower phase that contained PVP-bonded tannins was separated from the upper phase. Folin-Ciocalteau reagent and sodium-carbonate solution were added to the lower phase and the mixture was kept at 40 ^°^C for 40 min. Finally, absorbance of the final solution was measured at 725 nm using a spectrophotometer (UNICO, USA). Dry weight of concentrated extract was determined by placing 1 ml of extract in a pre-weighed, clean, and dry disposable aluminum dish. Samples were dried in an oven at 40 ^°^C for 24 hr followed by cooling in a desiccator for at least 30 min prior to weighing. This procedure of drying and weighing was repeated three times to make sure that the sample was completely dry. The tannic acid solution in the range of 20–100 µg/ml was used as a control. 

***Preparation of green tea/polyvinyl alcohol formulation and rheological analysis***

The 7% w/v PVA solution was prepared by adding PVA powder to the cold water to avoid formation of lumps. Once the powder was fully dispersed, the mixture was heated and stirred at 60^ °^C for 3 hr. The hydroalcoholic extract of green tea was mixed with PVA solution to reach the final concentration of 2% and 4 % v/v and stirred to obtain a homogeneous solution. The viscosity of the solutions was measured using a Brookfield R/S+Rheometer (Brookfield Co., USA) rotational rheometer with the CC25 spindle at a shear rate of 0–200 sec ^−1^.

***Microbiological testing***

Microbiological experiments were conducted to determine the presence, number (quantitative test), and type (qualitative test) of microorganisms in raw materials and the final product (19). The pour plate method was used for quantitative test. Briefly, 1:10 and 1:100 dilutions of samples were prepared in SCDB using the serial dilution technique. One milliliter of each dilution was poured into an empty petri dish followed by adding the molten agar medium (SCDA, 45–50 ^°^C). The petri dishes were gently rotated to mix the culture medium and diluted samples thoroughly and incubated in an inverted position at 37 ^°^C for 2 days. At the end, the number of colonies were counted and reported.

In qualitative tests, absence of specific microorganisms including *Staphylococcus aureus*,* Pseudomonas aeruginosa*,* Salmonella *spp.*, Escherichia coli*, and *Candida albicans *were tested. From the SCDB culture of the green tea/PVA 4% v/v was cultured on a petri dish or in a tube of specific culture medium including Vogel-Johnson agar for *S. aureus*, cetrimide agar for *P. auroginosa*, tetrathionate broth, and then bismut sulfite agar for *Salmonella* spp., and MacConkey agar for *E. coli*, and Sabouraud dextrose broth for *C. albicans*. The plates and tubes were incubated at 37 ^°^C for 24 to 48 hr and then all culture media were carefully observed to detect the absence of specific microorganisms (20). 

***Determination of minimum inhibitory concentration (MIC)***

The antimicrobial effect of green tea extract (4% v/v), PVA, and green tea/PVA 4% v/v on *S. aureus and P. aeruginosa* was evaluated by MIC assay (21-23). In brief, the solutions were serially diluted (at a ratio of 1:3) to make a final concentration between 22 to 11500 µg/ml and poured into 96-well plates. Then, approximately 10^6^ CFU/ml of overnight culture of *S. aureus *or* P. aeruginosa* was seeded into each well containing SCDB and each sample. The plates were incubated for 24 hr at 37 ^°^C and MIC was determined as the lowest concentration of the samples that inhibited the growth of bacteria. 

***Evaluation of the time to achieve hemostasis***

The protocol of animal study was approved by the Local Ethics Committee of the Mashhad University of Medical Sciences (IR.mums.ac.1396.178). In order to determine the effect of green tea extract on achieving hemostasis, mice with about 30 g weight and 10 weeks’ age were divided into 16 groups. Each group contained 5 mice. On the day of experiment, the mice were anesthetized and their tails were disinfected and cut oﬀ at a distance of 2.5 cm from the tail end. Immediately after, the cut tail was placed inside a tube containing 10 ml of test solutions. The time taken to achieve hemostasis was measured using a chronometer. Phosphate buffered saline (PBS) pH 7.4 and tranexamic acid 10–50 mg/ml were used as negative and positive controls, respectively. As another positive control, direct finger pressure on the cutting place was applied for 10 and 20 sec (24).

***Statistical analysis***

The results of 5 replicates were reported as mean ± SEM. Statistical analyses were done using the IBM SPSS Statistics 20 software package. To evaluate differences between groups, one-way analysis of variance (ANOVA) was used followed by the Tukey-Kramer *post hoc* test. *P*-value˂0.05 was considered statistically significant.

## Results

***Total tannins content in the green tea extract***

**Figure 1:**
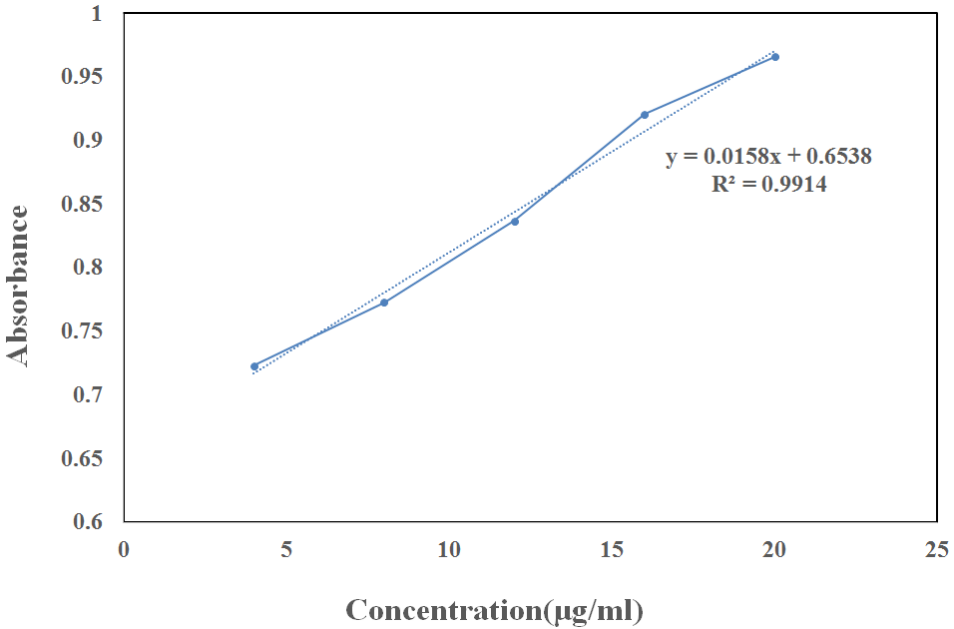
Standard calibration curve of tannic acid

Total tannins in hydroalcoholic extract of green tea were determined by Folin-Ciocalteau assay. The standard curve of tannic acid solution in the concentration range of 20–100 µg/ml is shown in Figure 1. The absorbance of green tea extract was about 0.869, which was equivalent to 13.62 μg/ml according to the standard curve. The dry weight of 1 ml of concentrated extract was 8.6 mg. So, the amount of tannin in 1 ml of green tea extract was 326.88 μg, which was equal to 3.8 % w/w. 

***Viscosity of PVA and green tea/PVA formulations***

**Figure 2:**
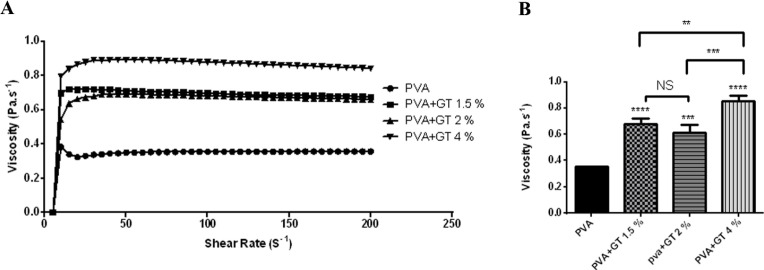
A: Viscosity as a function of shear rate (sec^-1^) for Polyvinyl alcohol (PVA) solution and PVA+ green tea extract (GT) solutions with different GT concentrations. B: Apparent viscosity of PVA solution and PVA+ green tea (GT) solutions with different GT concentrations. Data represent the average of all 3 experimental replicates and the error bars represent SD. (Pa.S: The SI derived unit for dynamic viscosity is the pascal-second. 1 pascal/second is equal to 1000 centipoise)

The rheological behavior of PVA solution and green tea/PVA formulations is exhibited in Figure 2. As shown in Figure 2A, the flow properties of PVA and all green tea solutions were rather close to those of Newtonian fluids. The viscosity of Newtonian fluids remains constant in different shear rates. Figure 2B shows the apparent viscosity of PVA and different green tea solutions. The results showed that adding green tea extract increased the viscosity and this rise was more significant in 4% v/v solution (0.85±0.043 Pa.sec^-1^ compared with 0.36 ± 0.00 Pa.sec^-1^ for PVA solution without green tea) .Both PVA and polyphenols in green tea have a hydroxyl group in their structure which can participate in hydrogen bonding (25, 26). Significant increase in viscosity of solutions after adding green tea extract is believed to be due to the interaction between PVA and green tea through formation of hydrogen bonds.

***The results of microbiological testing and antibacterial activity***

In quantitative microbiological test, microbial growth was observed just in the plates that contained 1:10 dilution of extract, but the number of colonies was less than 7, which is lower than the limit according to the U.S. Pharmacopeia-National Formulary. The results of qualitative test showed that no microorganism growth was observed in any specific culture media for *S. aureus, P. aeruginosa, Salmonella* spp.,* E. coli, and* *C. albicans. *

The results of MIC assays showed that PVA alone could not inhibit the growth of *S. aureus* and *P. aeruginosa. *The MIC of green tea extract 4% and green tea/PVA 4% v/v on *S. aureus* was the same (716 µg/ml), while it was different on *P. aeruginosa* and decreased from 5730 µg/ml in green tea 4% to 2860 µg/ml when formulated in green tea/PVA 4% formulation. MIC of amoxicillin as a positive control for *S. aureus* was 8 μg/ml and MIC of streptomycin as a positive control for *P. aeruginosa* was 120 μg/ml.

***Evaluation of the time to achieve hemostasis***

**Figure 3:**
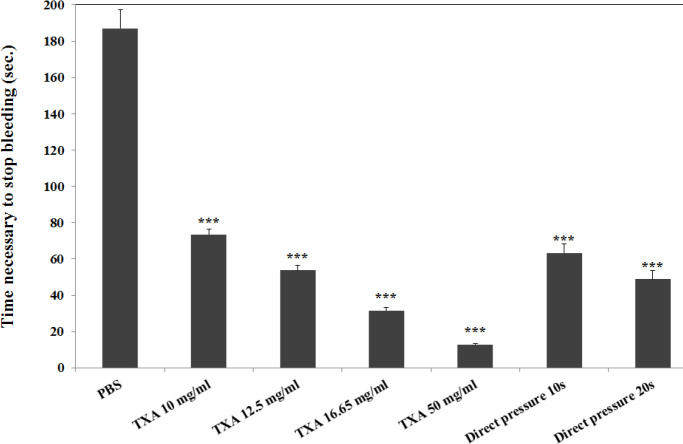
Comparison of time to achieve hemostasis after cutting off rat tail in different concentrations of tranexamic acid (TXA) and direct finger pressure (direct pressure) as a positive control and phosphate buffered saline (PBS) as negative control groups. In each group, 5 mice were evaluated and the results were reported based on mean±SEM. ***: *P*-value <0.001 vs PBS group

The effect of PVA, green tea extracts, and green tea/PVA formulation on bleeding time was investigated on the mouse tail. According to the results, the longest bleeding time was 187±42.7 sec for BPS (negative control), which was significantly higher compared with all other groups (*P*-value=0.00). Tranexamic acid solutions (positive control) significantly controlled the bleeding in a concentration dependent manner. The maximum inhibitory effect on hemorrhage in control groups was observed in 50 mg/ml of tranexamic acid with bleeding time of 12.8±0.66 sec (Figure 3). 

**Figure 4:**
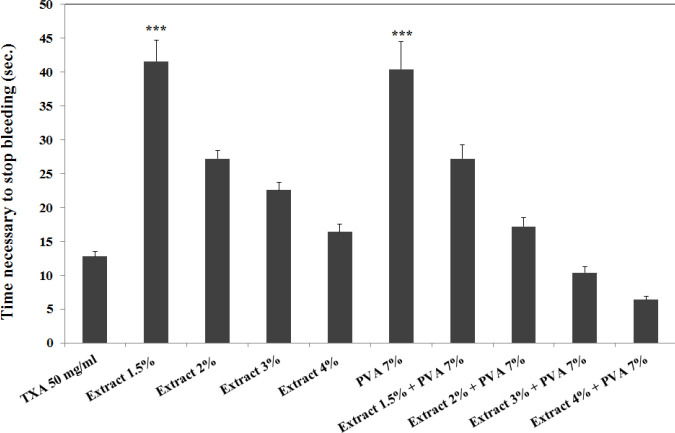
The effect of different concentrations of green tea extract (extract) alone or in combination with PVA to stop bleeding in rat tail. In each group, 5 mice were evaluated and the results were reported based on mean±SEM. ***: *P*-value <0.001 vs tranexamic acid (TXA) 50 mg/ml group

Green tea extracts 2% and 4% were able to stop bleeding approximately equal to tranexamic acid 50 mg/ml. The results showed that the bleeding time in the green tea/PVA groups decreased up to 11.66 sec compared with the extract group at the same concentration. The lowest bleeding time was 6.4±0.51 sec, which was observed in green tea/PVA 4% (v/v) (Figure 4). 

## Discussion

Uncontrolled bleeding is a major cause of preventable death from severe traumatic injury or vascular surgery (1-3). Therefore, it is very important to use different techniques, devices, and drugs for rapid and sufficient control of hemorrhage (2, 3). Green tea has been consumed for its health benefits all over the world for centuries, but recently because of its great properties such as anticoagulant, antibacterial, antiviral, and antiseptic effects, it has received much attention for topical usage (4, 5, 27, 28). In this study, the ability of green tea extract to control hemorrhage was studied and an effective topical formulation of green tea extract in PVA was designed to improve its hemostatic and antimicrobial affects.

The results of our study showed that green tea extract was able to inhibit hemorrhage much more potently than positive control groups and almost equal to the strongest positive control group (tranexamic acid 50 mg/ml). Few studies have been done on effect of green tea on bleeding control mostly in the field of oral and dental care. In one study, sterile gauze impregnated with green tea extract was directly applied for stopping gingival bleeding after tooth extraction. The results indicated that green tea extract significantly decreased bleeding to about two-fold compared with the control group (extract-free gauze) (27). 

Tea, whether it’s black, green, or any other color contains tannins which provide astringent properties. These tea components can accelerate blood clotting and stop bleeding (10, 29). In our study, 3.8% (w/w) of green tea extract consisted of tannins. Khasnabis *et al.* reported the same result when they studied the tannin content of various types of green tea. They reported that the highest tannin content was about 3.11% while the average was 2.65% (30). The higher amount of tannins in green tea could be beneficial because it seems that tannins are responsible for the effects of green tea on blood clothing and control of bleeding. 

Our results showed that the lowest bleeding time (6.4±0.51 sec) was observed in green tea/PVA 4% (v/v). This would indicate that the formulation of green tea extract in PVA not only kept green tea’s bleeding control effect but also shortened the bleeding time even more than extract alone. PVA, as another part of the formulation, was chosen due to its suitable properties like non-toxicity, water solubility, extensive use in drug delivery systems, natural adhesion, and viscosity enhancement properties (14, 31). PVA alone was able to stop bleeding approximately equal to one of our positive controls (tranexamic acid 12.5 mg/ml). Investigating the rheological behavior of PVA and green tea/PVA solutions revealed that the combination of different concentrations of extract and PVA significantly increased the viscosity. Therefore, it could be hypothesized that PVA increased the effect of green tea on the control of bleeding by increasing the viscosity and holding the extract longer at the site of injury. 

In microbiological testing, no important microorganism growth was observed, indicating that green tea/PVA products meet the microbial limit test of the US Pharmacopeia. On the other hand, the MIC assays showed that green tea extract and green tea/PVA formulations had antimicrobial effects against Gram-positive and Gram-negative bacteria (MIC 716 µg/ml against* S. aureus, *and MIC 2860 µg/ml against* P. aeruginosa). *In similar studies, the MIC of green tea extracts against *S. aureus* and *P. aeruginosa* was reported between 300–1000 µg/ml (19, 23). It seems that the difference between MICs results from difference in some criteria like plant source, microbial strain, and extract solvent. 

The antimicrobial activities of tannins are well documented. These antimicrobial effects have been shown to be related to catechins (32). The growth of many Gram-positive and Gram-negative bacteria, fungi, and viruses was inhibited by green tea catechins (19, 32). In some studies, green tea was formulated as a mouthwash and the effect of it on oral health was investigated. Their results showed green tea mouthwash could significantly reduce plaque index, gingival index, and bleeding on probing without causing any side effects (33). The ability of green tea mouthwash to improve oral health results through antimicrobial activity of green tea catechins has been reported to be due to different mechanisms such as damaging bacterial lipid bilayer cell membrane, inhibiting enzyme activity, and inhibiting fatty acid synthesis (32). 

## Conclusion

The results of this study showed that green tea/PVA formulation significantly reduced bleeding time. Moreover, it showed considerable antimicrobial effects that can play an important role in preventing secondary infections. Based on the good results in animal studies and microbial tests, ease of access, and low price, green tea/PVA formulation has a great potential for use in bleeding control. 
